# Recognition memory of neutral words can be impaired by task-irrelevant emotional encoding contexts: behavioral and electrophysiological evidence

**DOI:** 10.3389/fnhum.2015.00073

**Published:** 2015-02-13

**Authors:** Qin Zhang, Xuan Liu, Wei An, Yang Yang, Yinan Wang

**Affiliations:** ^1^Learning and Cognition Key Laboratory of Beijing, Department of Psychology, Capital Normal UniversityBeijing, China; ^2^Department of Psychology, University of NevadaLas Vegas, NV, USA; ^3^Mental Health Education Center, Huanghuai UniversityZhumadian, China

**Keywords:** emotional context, neutral words, recognition, event-related potential (ERP), familiarity, recollection

## Abstract

Previous studies on the effects of emotional context on memory for centrally presented neutral items have obtained inconsistent results. And in most of those studies subjects were asked to either make a connection between the item and the context at study or retrieve both the item and the context. When no response for the contexts is required, how emotional contexts influence memory for neutral items is still unclear. Thus, the present study attempted to investigate the influences of four types of emotional picture contexts on recognition memory of neutral words using both behavioral and event-related potential (ERP) measurements. During study, words were superimposed centrally onto emotional contexts, and subjects were asked to just remember the words. During test, both studied and new words were presented without the emotional contexts and subjects had to make “old/new” judgments for those words. The results revealed that, compared with the neutral context, the negative contexts and positive high-arousing context impaired recognition of words. ERP results at encoding demonstrated that, compared with items presented in the neutral context, items in the positive and negative high-arousing contexts elicited more positive ERPs, which probably reflects an automatic process of attention capturing of high-arousing context as well as a conscious and effortful process of overcoming the interference of high-arousing context. During retrieval, significant FN400 old/new effects occurred in conditions of the negative low-arousing, positive, and neutral contexts but not in the negative high-arousing condition. Significant LPC old/new effects occurred in all conditions of context. However, the LPC old/new effect in the negative high-arousing condition was smaller than that in the positive high-arousing and low-arousing conditions. These results suggest that emotional context might influence both the familiarity and recollection processes.

## Introduction

The question that how emotion influences human memory has drawn a lot of attention and many studies have demonstrated that emotional items or events are more likely to be remembered than neutral items or events (Dolan, 2002; LaBar and Cabeza, 2006). This memory advantage might be attributed to enhanced attention and elaboration (Koening and Mecklinger, 2008) as well as strengthened consolidation of memory traces (Talmi, 2013).

However, when considering the effects of emotion on memory for items presented in the context of other stimuli, some studies found emotion-induced impairment rather than enhancement on memory performance (see Chiu et al., 2013). And this memory impairment has been linked to two factors. One is the type of the information tested. When testing memory for contextual information or relational binding information between contexts and items or between item pairs, impairing effects of emotion on memory might be observed (see Chiu et al., 2013). For example, Kensinger et al. (2007) found that memory for scene contexts accompanying emotional items was less detailed. Rimmele et al. (2011) found lower recognition accuracy for frame colors surrounding negative scenes than for frame colors surrounding neutral scenes. Chiu et al. (2013) proposed that when contextual/relational information and item information cannot be represented in a unitized manner, there might be emotion-induced impairment on memory for contextual/relational information. Another factor is the presentation position of the information tested. According to the hypothesis of emotional trade-off effect on memory for central vs. peripheral information, emotion can enhance memory for central information but impair memory for peripheral information. Some findings supported this hypothesis. For example, Touryan et al. (2007) showed impaired memory for objects that were peripheral to negative emotional pictures. Kim et al. (2013) also found that memory performance for peripheral items occurring in negative picture stories was worse compared to neutral picture stories.

Emotion impairs memory for contextual/relational information or peripheral information. Then, is memory for central to-be-remembered items always enhanced by emotion? Previous studies obtained inconsistent results on the role of emotional contexts on memory for centrally presented neutral items. Smith et al. (2004) used a scene context design to investigate how emotional backgrounds (negative, positive, or neutral pictures) affect memory for neutral objects superimposed centrally onto them. The results showed that items associated with positive contexts were recognized more accurately than those encoded in either neutral or negative contexts. However, employing the similar design, other studies did not find memory enhancement for items centrally presented in the emotional contexts (Erk et al., 2005; Jaeger et al., 2009). Using a serial presentation design, Anderson et al. (2006) found that emotional pictures enhanced memory for neutral pictures preceding them. Other studies, however, showed impaired memory for items preceding or following an emotional item (e.g., Strange et al., 2003; Hurlemann et al., 2005). Knight and Mather (2009) demonstrated both emotion-induced enhancement and impairment. They suggested that emotion-induced memory impairment for neutral items is most likely to occur near the onset of emotional arousal. In contrast, when neutral items precede emotional items and have high attentional weights during encoding, emotion-induced enhancement for neutral items is most likely to occur.

Some obvious differences in terms of the experimental design between studies using the scene context design and studies using the serial presentation design should be responsible for the inconsistent results mentioned above. In the study of Smith et al. (2004), a neutral object was superimposed centrally onto the background in the study phase, and participants were asked to imagine a connection between the object and the background. This instruction encouraged participants to represent the item and the context in a unitized manner. Under this circumstance, emotion-induced enhancement of memory is likely to be observed (Chiu et al., 2013). In contrast, in the studies using the serial presentation design, emotional and neutral items were presented sequentially and all stimuli were asked to be remembered. Therefore, interference between different stimuli might be high and emotional stimuli can consume cognitive resources that might otherwise be devoted to rehearsing the neutral items (Mather et al., 2006). As a result, emotion-induced impairment of memory is most likely to be observed.

In contrast to previous studies, the present study examined the role of emotional backgrounds on memory for neutral items using a scene context design where participants were neither asked to make a connection between the item and the background nor asked to remember the background. Because no response for contexts was required, participants would try their best to ignore contexts. Then, how do emotional stimuli presented only as task-irrelevant study context influence memory for task-relevant neutral items? Because emotional contexts might still capture participants' attention and influence perceptual processing and encoding of test items (Müller et al., 2008; Bocanegra and Zeelenberg, 2009), it is possible that emotional contexts would impair recognition performance for these neutral items. Moreover, unlike Smith et al. (2004) study in which the emotional background was initially presented alone on the screen for several seconds and then the neutral item was superimposed centrally onto the background, in the current experiment the emotional backgrounds and the neutral items were presented simultaneously and thus there is more likely to be interference of contexts for items. In addition, previous studies mainly adopted two types of emotional contexts- negative and positive-to investigate effects of emotional (vs. neutral) contexts on memory. Given that the main dimensions of emotion include both valence and arousal (Lang et al., 1993) and emotional contexts can be classified more carefully, the present study employed four types of emotional contexts including negative low-arousing, negative high-arousing, positive low-arousing, and positive high-arousing contexts to explore how different emotional pictures as encoding contexts would influence memory for neutral words. We hope to further clarify the circumstances under which emotion-induced impairment is likely to occur and gain insight into the mechanisms of emotion-induced memory impairment.

Besides behavioral measurements, the present study also used event-related potential (ERP) measurements to investigate the memory processes of neutral words encoded in different emotional contexts. So far a number of studies have examined the processing of emotional stimuli employing ERPs and showed that emotion can modulate ERP amplitudes (e.g., Schupp et al., 2000; Keil et al., 2002; Delplanque et al., 2004; Carretie et al., 2006). The earlier ERP components indexing emotion effects (<300 ms) have been theoretically linked to attention orientation for unpleasant pictures and the later ERP components (>300 ms) have been considered to reflect mental resource allocation and memory formation (see Olofsson et al., 2008). In the present study, because neutral words were superimposed onto emotional pictures during encoding, ERP measurements at study should reflect the processing of both neutral words and emotional pictures. We expected to observe strong emotional effects on memory encoding in the earlier and later ERP components.

Compared to encoding, the current study is more interested in effects of emotion on memory retrieval which involves two distinct processes (Mandler, 1980; Jacoby and Dallas, 1981; Jacoby, 1991; Yonelinas, 2002; Rugg and Curran, 2007). The first process is familiarity, which occurs when people feel that an item is familiar but cannot recall any detailed information. The second process, recollection, occurs when people can recall an item along with the details of the encoding episode. ERP studies on recognition memory have identified two old/new effects (in which ERP waveforms elicited by correctly classified “old” items are more positive than waveforms elicited by correctly classified “new” items) that are considered to index familiarity and recollection, respectively. The earlier mid-frontal old/new effect (FN400 that peaked around 400 ms) is attributed to familiarity (Curran, 2000; Rugg and Curran, 2007). However, some researchers have suggested that FN400 is not the ERP index of familiarity but instead indicates conceptual priming (Paller et al., 2007; Hou et al., 2013). The parietal old/new effect representing recollection is reflected by a late positive component (LPC) peaking at about 600 ms (Curran et al., 2006; Rugg and Curran, 2007; Woroch and Gonsalves, 2010). In addition, a late post-retrieval old/new effect reflected by a late slow wave (LSW) in the frontal area seems to index an ensemble of evaluation processes, and it is interpreted as the monitoring of the retrieved information (Rugg et al., 1998; Rugg and Allan, 2000; Rugg and Wilding, 2000).

An interesting question is which ERP component, FN400, LPC, or LSW, might be influenced by emotional encoding contexts. Previous research on emotional context effects obtained different ERP results. Maratos and Rugg (2001) examined ERP correlates of the retrieval of words presented in negative and neutral sentences and found that the left parietal ERP old/new effect was greater for negative than for neutral contexts during 500–1100 ms post-stimulus time window, which suggests that recognition of words encoded in negative sentences was associated with the retrieval of more information about the encoding episode than recognition of the words encoded in neutral sentences. They also found that the right frontal old/new effect was elicited solely by words studied in negative sentences during 1100–1944 ms, reflecting post-retrieval processing such as evaluation and monitoring operations. Smith et al. (2004) found that the most evident ERP effects of emotional (negative and positive) context (vs. neutral context) emerged around 800 ms post-stimulus. They argued that the relatively late emotion effects reflected an emotional modulation of post-retrieval processing. Because the explicit retrieval for the encoding context was unnecessary in this experiment as well as in the experiment by Maratos and Rugg (2001), the effects of emotion should reflect incidental retrieval and subsequent processing of contextual information (Maratos and Rugg, 2001; Smith et al., 2004). Additionally, Smith et al. (2004) found a small effect of emotional context in the 300–500 ms time window and proposed that this earlier effect indicated that items paired with emotional contexts at study had acquired some of the emotional attributes of these contexts and did not depend upon conscious recollection of the contexts. Employing the similar procedure, Jaeger et al. (2009) investigated ERP correlates of the retrieval of neutral objects paired with negative or neutral scenes at study over two study-test delays (10 min vs. 24 h) and found a sustained positive-going modulation in the ERPs elicited by objects paired with negative relative to neutral contexts from around 200 ms post-stimulus at the short delay. However, the ERP emotion effects at the long delay differed from those observed at the short delay in terms of both polarity and scalp distribution. The authors proposed that the influence of emotional context on retrieval might be modulated by emotionally-specific consolidation mechanisms.

In the present study, we also attempted to determine which ERP component might be influenced by emotional encoding contexts and thus infer whether and how emotional contexts affect familiarity, recollection, or post-retrieval process. Based on previous ERP research as well as the view that familiarity process is rather automatic whereas recollection is conscious retrieval of details associated with a previously experienced event (Curran, 2000; Curran et al., 2006; Rugg and Curran, 2007), we predicted that emotional contexts would modulate LPC and LSW old/new effects.

In sum, the present study aimed at exploring the influence of emotional picture contexts on memory for neutral words with both behavioral and ERP measurements. Given the evidence that high-arousing stimuli can capture attention more effectively than low-arousing and neutral stimuli (Dolcos and Cabeza, 2002; Schupp et al., 2003) and that negative stimuli can attract attention more effectively than positive stimuli in the early stage of visual processing (Delplanque et al., 2004; Carretie et al., 2006), we expected to find emotion-induced memory impairment for neutral words under negative or high-arousing contexts. Given previous ERP studies, we also expected that strong emotional effects in the earlier and later ERP components would be observed at study and that emotional contexts might modulate LPC and LSW old/new effect at retrieval.

## Materials and methods

### Participants

Fourteen right-handed undergraduate students (ranging in age from 20 to 25, 7 male and 7 female) participated in the experiment. All participants were native Chinese speakers and had normal or corrected-to-normal vision. Informed consent was obtained from each participant before his/her participation. This research was approved by the Institutional Review Board of the Capital Normal University.

### Stimuli

Two hundred and fifty pictures were selected as encoding contexts from the International Affective Picture System [IAPS, Lang et al. (1999)], which were divided into 5 groups on the base of valence and arousal: negative low-arousing pictures, negative high-arousing pictures, positive low-arousing pictures, positive high-arousing pictures, and neutral pictures (IAPS number of these pictures were shown in the Supplementary Material). Each group contained 50 pictures, with mean valence and arousal shown in Table 1. The valence and arousal differences between neutral pictures and any other type of pictures were significant (*p*s < 0.05). The valence difference between positive and negative pictures was significant (*p* < 0.05). The arousal difference between high-arousal and low-arousal pictures was significant (*p* < 0.05). However, the valence difference between high-arousal and low-arousal pictures and the arousal difference between positive and negative pictures also were significant (*p*s < 0.05).

**Table 1:** **Mean (*SD*) valence and arousal of five types of pictures**.

**Types of pictures**	**Valence (*SD*)**	**Arousal (*SD*)**
Negative low-arousal	3.13 (0.51)	4.53 (0.47)
Negative high-arousal	2.12 (0.58)	6.45 (0.61)
Positive low-arousal	7.47 (0.40)	4.13 (0.57)
Positive high-arousal	6.45 (0.65)	5.92 (0.70)
Neutral	5.06 (0.38)	3.04 (0.73)

Five hundred neutral words (mean valence = 5.3, ranging from 3.65 to 6.5) were chosen from the Chinese Affective Words System [CAWS, Luo and Wang (2004)]. Two hundred fifty words were paired with 250 pictures, as target words and encoding contexts during the learning phase. Another 250 words as new words, together with the 250 learned words, were used in the test phase. Mean valence, arousal, and familiarity of six groups of words were shown in Table 2. There was no significant valence, arousal, or familiarity difference among six groups of words (*p*s > 0.05).

**Table 2:** **Mean (*SD*) valence, arousal, and familiarity of six groups of words**.

	**Valence (*SD*)**	**Arousal (*SD*)**	**Familiarity (*SD*)**
Words paired with negative low-arousal contexts	5.27 (0.89)	4.57 (0.66)	5.24 (0.51)
Words paired with negative high-arousal contexts	5.36 (0.81)	4.62 (0.78)	5.10 (0.52)
Words paired with positive low-arousal contexts	5.48 (0.80)	4.63 (0.64)	5.04 (0.54)
Words paired with positive high-arousal contexts	5.14 (0.82)	4.53 (0.54)	5.30 (0.44)
Words paired with neutral contexts	5.41 (0.77)	4.53 (0.80)	5.27 (0.49)
New words	5.28 (0.84)	4.60 (0.64)	5.19 (0.52)

Besides the pictures and words mentioned above, we chose another 20 pictures from IAPS, paired with 20 city names, as filler stimuli during the learning phase. All stimuli were assigned to 18 lists. Each list contained 15 word-picture pairs (0–3 pairs being filler stimuli and others being target word—picture context pairs) that would be presented during the learning phase. All target words and an equal number of new words would be presented during the test phase.

### Procedure

**Figure 1:**
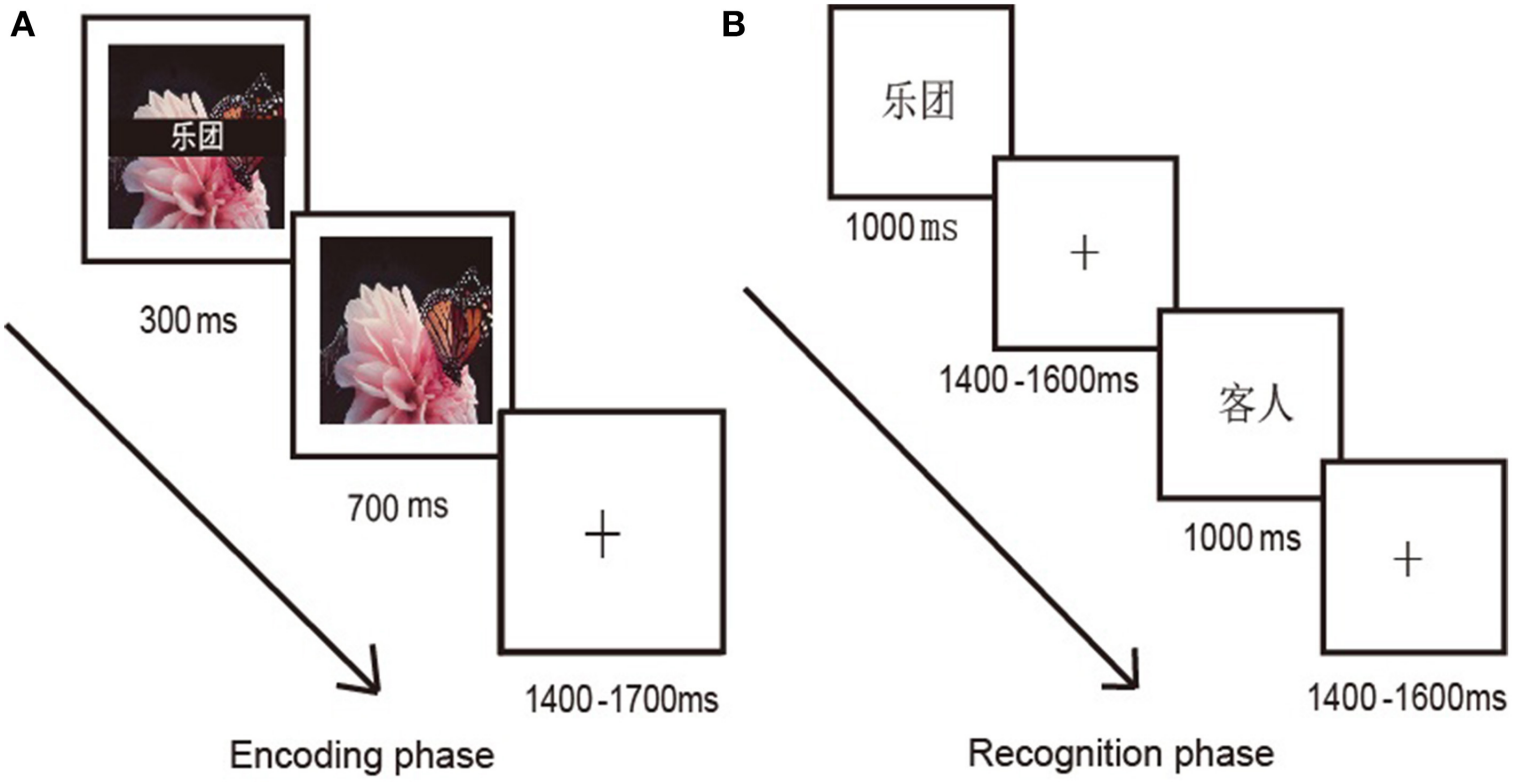
**Sample stimuli and procedures for encoding (A) and recognition phase (B)**.

The present study used a classical study-test paradigm. Each of the 18 study-test runs included an encoding phase, a distraction phase, and a recognition phase. During encoding, each trial began with a word-picture pair that was displayed on the screen. The word was superimposed onto the central part of the picture (see Figure 1). After 300 ms, the word disappeared and the picture remained on the screen for another 700 ms. Following the disappearing picture was a fixation cross presented for 1400–1700 ms, and then the next trial started. Participants were instructed to just remember the word, and simultaneously press the key quickly and accurately as soon as they saw the city name. The distraction task was subtraction beginning with a 3-digit number shown on the screen. Participants repetitively subtracted 3 from each number (e.g., 675: 672, 669, 666,…) for 60 s. In the recognition phase, each word was displayed on the screen for 1000 ms and interstimulus interval (ISI) was 1400–1600 ms. Participants were required to press one of two buttons to indicate whether the displayed target was a new word or an old word (i.e., a word from the prior encoding phase). The assignment of response keys to left/right hand was counterbalanced across subjects. Participants were also instructed to respond as quickly and accurately as possible, and to refrain from head movements and eye blinking. Stimuli were presented in pseudo-random order during the encoding and recognition phases.

### Records and analysis of ERPs

EEG recordings were made from 64 scalp sites using Ag/AgCl electrodes arranged in an elastic cap. Two pairs of electrodes were used for monitoring vertical and horizontal eye movements. A left mastoid reference electrode was used online and the reference was changed offline to the average of left and right mastoid recordings. EEG signals were filtered with a bandpass of 0.05 ~ 40 Hz and sampled at a rate of 500 Hz. Impedances were kept below 5 KΩ. Average ERPs were formed offline from correct-response trials free of ocular and movement artifacts (> ±75 μV). Each averaging epoch lasted 1100 ms, including 100 ms prior to stimulus onset.

## Results

### Behavioral results

Table 3 displays the recognition accuracies and reaction times (RTs) of new words and old words learned in different contexts. A one-way (experimental condition: negative high-arousing, negative low-arousing, positive high-arousing, positive low-arousing, neutral context, new words) repeated-measures analysis of variance (ANOVA) for RTs showed that the condition effect did not reach significance [*F*_(5, 65)_ = 1.50, *p* > 0.20]. The one-way repeated-measures ANOVA for accuracies indicated a significant condition effect [*F*_(5, 65)_ =15.36, *p* < 0.001, η^2^_*p*_ = 0.542]. The multiple comparisons showed that participants' responses were more accurate for correctly rejected new words than for any type of correctly recognized old words (*p*s < 0.01). Accuracy was higher for words encoded in the neutral context than for words encoded in the negative high-arousing (*p* < 0.001), negative low-arousing (*p* < 0.05), or positive high-arousing context (*p* < 0.01). However, the difference between old words encoded in the neutral context and old words encoded in the positive low-arousing context did not reach significance (*p* > 0.30). The multiple comparisons also showed that participants' responses were more accurate for words learned in the positive low-arousing context than those in the negative high-arousing (*p* < 0.005) or positive high-arousing context (*p* < 0.05).

**Table 3:** **Mean accuracies and RTs (ms) of new words and old words encoded in different contexts**.

**Conditions**	**Accuracy (*SD*)**	**RT (*SD*)**
Negative low-arousal context	0.78 (0.13)	689 (77)
Negative high-arousal context	0.73 (0.10)	695 (74)
Positive low-arousal context	0.82 (0.07)	677 (83)
Positive high-arousal context	0.76 (0.11)	690 (65)
Neutral context	0.83 (0.09)	683 (70)
New words	0.93 (0.07)	705 (79)

### ERP results

#### Study-phase ERP results

**Figure 2:**
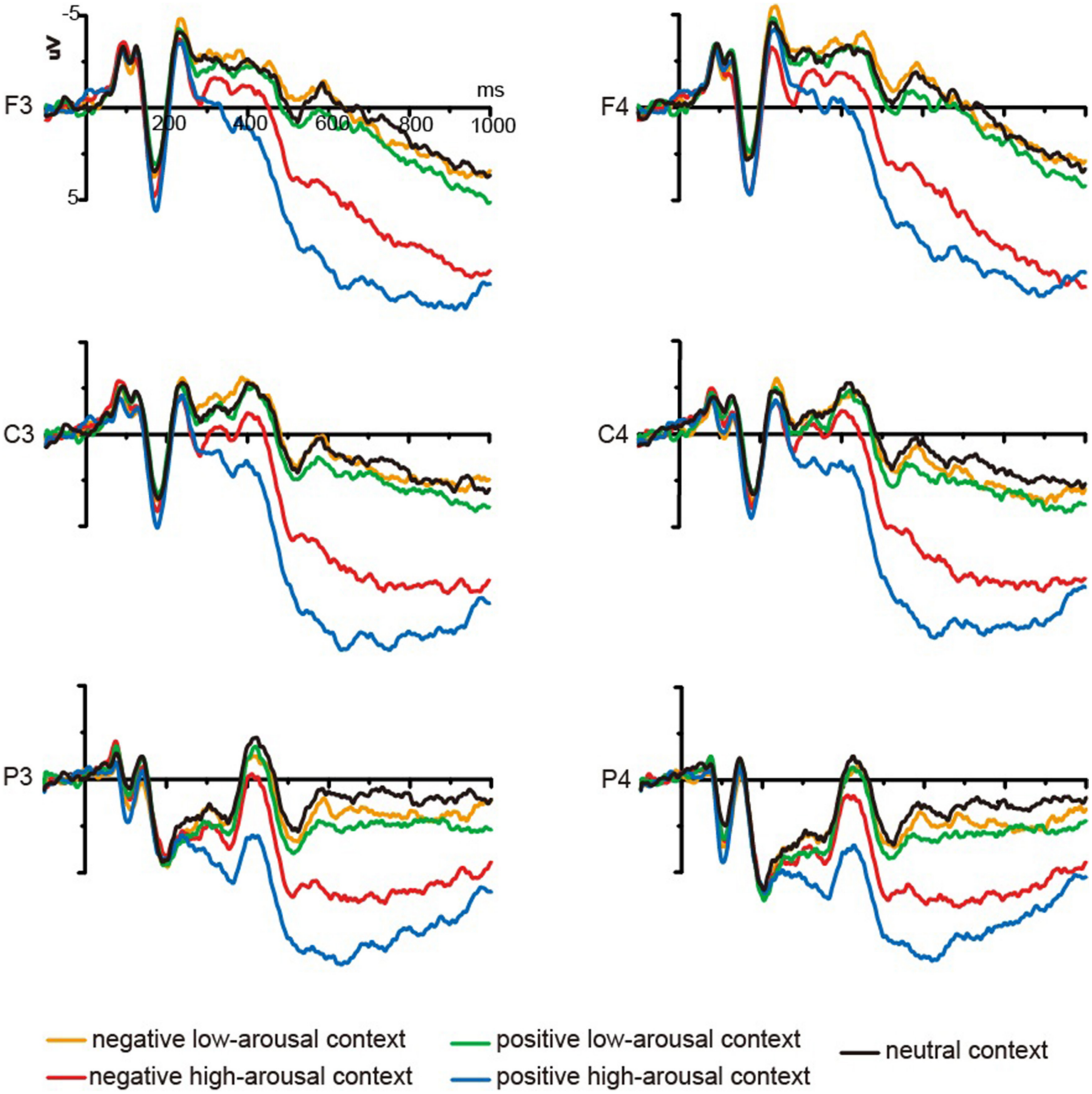
**Grand mean ERPs from F3, F4, C3, C4, P3, and P4 to words presented in five types of background pictures at encoding**.

Based on a careful examination of our grand average waveforms (see Figure 2) and a review of previous findings (see Olofsson et al., 2008), mean amplitudes were computed at three time windows (150 ~ 300 ms, 300 ~ 500 ms, and 500 ~ 1000 ms) for each subject and condition type. The amplitude measurements were referred to pre-stimulus baseline. The approach to statistical analysis involved the use of repeated-measures ANOVA and Greenhouse–Geisser corrections. The ANOVA was conducted by selecting 15 electrodes from left hemisphere, midline, and right hemisphere at frontal, fronto-central, central, centro-parietal, and parietal locations (F3, FZ, F4, FC3, FCZ, FC4, C3, CZ, C4, CP3, CPZ, CP4, P3, PZ, and P4). Five (condition: negative high-arousing, negative low-arousing, positive high-arousing, positive low-arousing, neutral context) × 5 (location: F, FC, C, CP, P) × 3 (laterality: left, midline, right) repeated-measures ANOVAs were conducted in three latency intervals. All significant main effects of condition or interactions between condition and other factor were supplemented with multiple comparisons or simple main effects comparisons when appropriate. In our ERP results, main effects of location and laterality and interaction of location and laterality were not reported.

***150–300 ms***. Our analyses showed a marginally significant main effect of condition [*F*_(4, 52)_ = 2.48, *p* = 0.073, η^2^_*p*_ = 0.160] and a significant interaction between condition and location [*F*_(16, 208)_ = 3.18, *p* = 0.015, η^2^_*p*_ = 0.197]. Further analyses showed that significant difference between neutral and negative high-arousing contexts occurred at frontal sites (*p* < 0.05). Significant difference between neutral and positive high-arousing contexts occurred at frontal and fronto-central sites (*p*s < 0.05). Moreover, significant differences between negative high-arousing and negative low-arousing contexts (*p*s < 0.05), between positive high-arousing and positive low-arousing contexts (*ps* < 0.05), and between negative high-arousing and positive low-arousing contexts (*ps* < 0.05) occurred at frontal and fronto-central sites. Significant differences between positive high-arousing and negative low-arousing contexts occurred at frontal, fronto-central, and central sites (*ps* < 0.05).

***300–500 ms***. Our analyses showed a significant main effect of condition [*F*_(4, 52)_ = 14.22, *p* < 0.001, η^2^_*p*_ = 0.522]. The multiple comparisons showed significant differences between neutral and negative high-arousing contexts (*p* < 0.05), between neutral and positive high-arousing contexts (*p* < 0.001), between negative high-arousing and negative low-arousing contexts (*p* < 0.05), between positive high-arousing and positive low-arousing contexts (*p* < 0.001), between positive high-arousing and negative low-arousing contexts (*p* < 0.001), and between positive high-arousing and negative high-arousing contexts (*p* < 0.05).

***500–1000 ms***. Our analyses showed a significant main effect of condition [*F*_(4, 52)_ = 39.52, *p* < 0.001, η^2^_*p*_ = 0.752] and significant interactions between condition and location [*F*_(16, 208)_ = 2.52, *p* = 0.049, η^2^_*p*_ = 0.162] and between condition and laterality [*F*_(16, 208)_ = 2.36, *p* = 0.049, η^2^_*p*_ = 0.154]. Further analyses showed that significant differences between neutral context and negative high-arousing context and between neutral context and positive high-arousing context occurred at all analyzed sites (*ps* < 0.001). In addition, significant differences between negative high-arousing and negative low-arousing contexts (*ps* < 0.001), between positive high-arousing and positive low-arousing contexts (*ps* < 0.001), between negative high-arousing and positive high-arousing contexts (*ps* < 0.05), between positive high-arousing and negative low-arousing contexts (*ps* < 0.001), and between negative high-arousing and positive low-arousing contexts (*ps* < 0.001) occurred at all analyzed sites.

#### Test-phase ERP results

**Figure 3:**
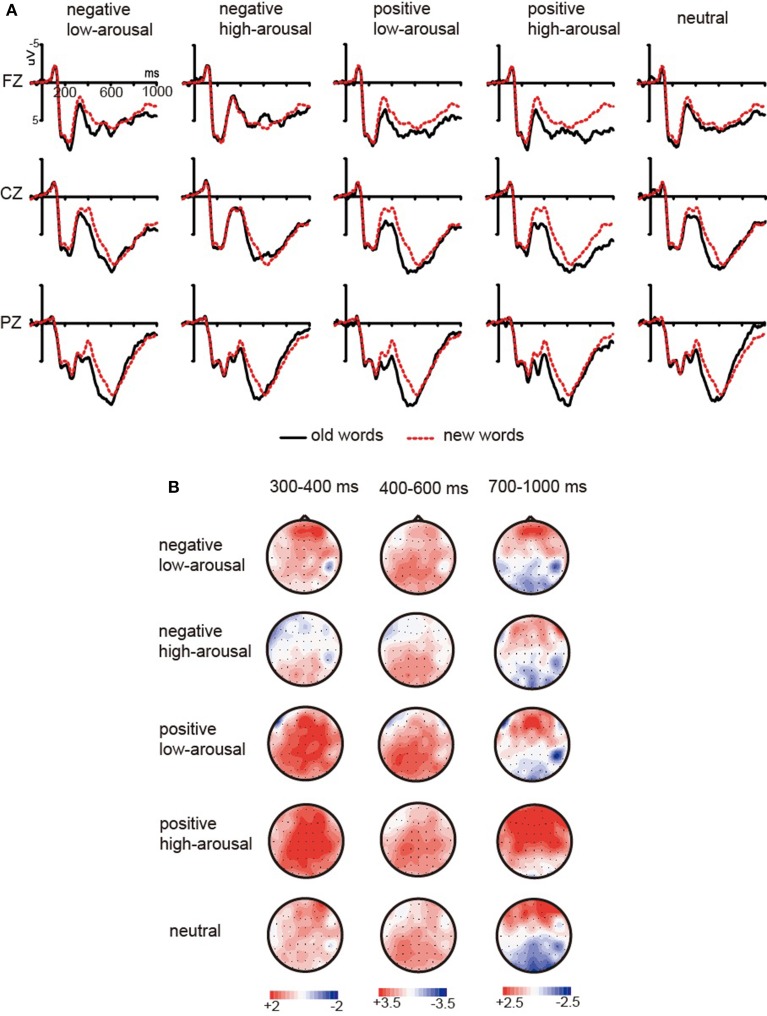
**(A)** ERP comparisons between new words and old words separately encoded in five types of background pictures across three scalp locations (Fz, Cz, Pz). Topographic maps **(B)** illustrate ERP difference waves computed by subtracting ERPs to new words from ERPs to old words across three intervals (300–400 ms, 400–600 ms, and 700–1000 ms).

**Figure 4:**
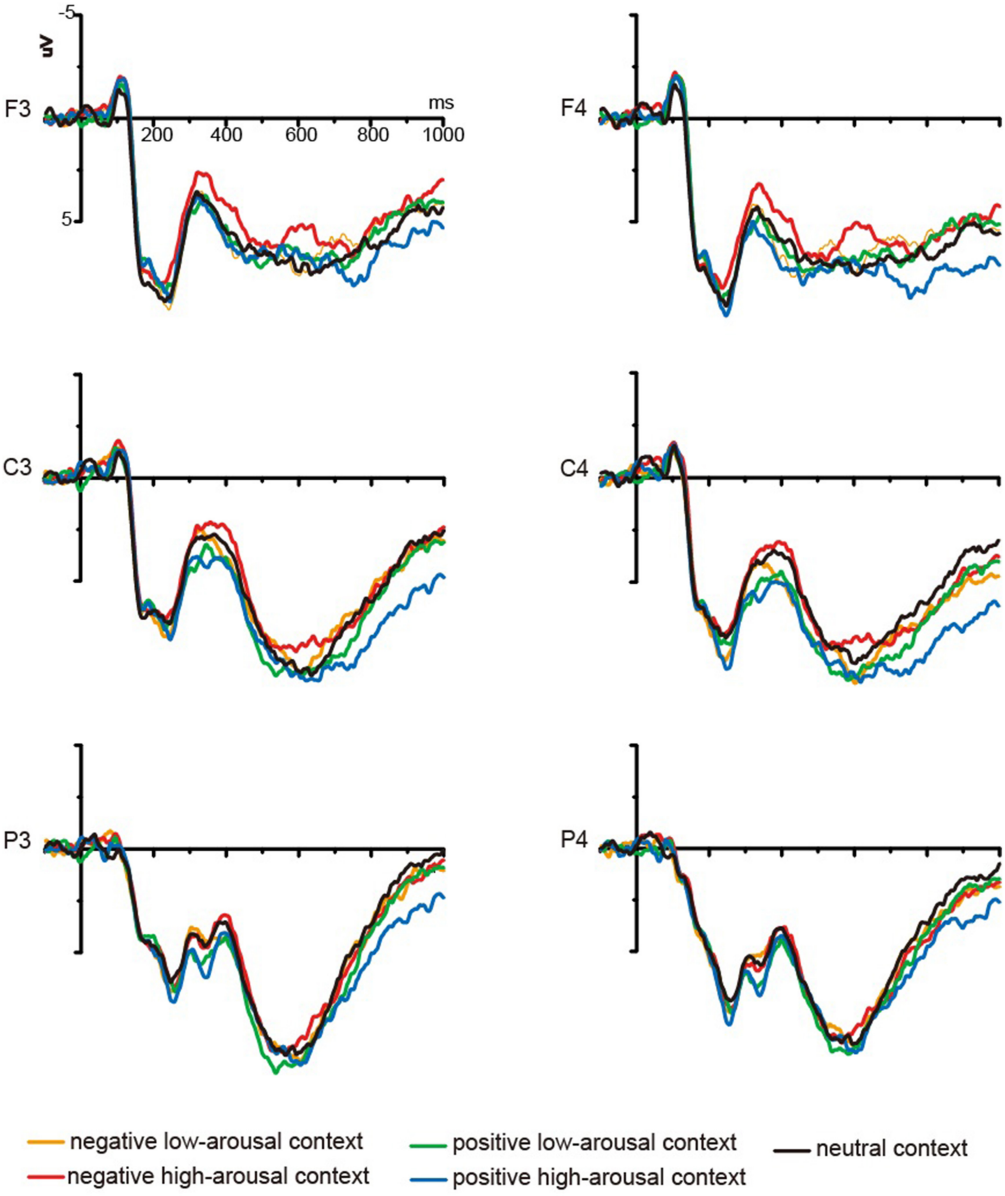
**Grand mean ERPs from F3, F4, C3, C4, P3, and P4 to words encoded in five types of background pictures during recognition**.

Grand average ERP waveforms at retrieval were shown in Figures 3, 4. A visual inspection of the waveforms was initially conducted to detect obvious differences among different encoding context conditions. The current study focused on whether FN400, LPC, and LSW might be influenced by emotional encoding contexts. FN400, LPC, and LSW were thought to onset around 300 ms, 400–500 ms, and 700–800 ms post-stimulus, respectively (Curran and Cleary, 2003; Smith et al., 2004; Rugg and Curran, 2007; Van Strien et al., 2009). Based on the timing of memory effects in previous studies and visual inspection of the present data, mean amplitudes were computed at time windows 300 ~ 400 ms, 400 ~ 600 ms, and 700 ~ 1000 ms for each subject and condition type. Six (condition: negative high-arousing, negative low-arousing, positive high-arousing, positive low-arousing, neutral context, new words) × 5 (location: F, FC, C, CP, P) × 3 (laterality: left, midline, right) repeated-measures ANOVAs were conducted in three latency intervals.

***300–400 ms***. Our analyses showed a significant main effect of condition [*F*_(5, 65)_ = 5.87, *p* = 0.001, η^2^_*p*_ = 0.311]. The multiple comparisons showed that ERPs elicited by new words were more negative than ERPs elicited by old words encoded in the negative low-arousing (*p* < 0.05), positive low-arousing (*p* < 0.005), positive high-arousing (*p* < 0.005), and neutral contexts (*p* < 0.05). However, the difference between new words and old words encoded in the negative high-arousing context did not reach significance (*p* > 0.10). Moreover, ERPs elicited by old words encoded in the negative high-arousing context were more negative than ERPs elicited by old words encoded in the positive high-arousing (*p* < 0.01) and positive low-arousing contexts (*p* < 0.005).

***400–600 ms***. Our analyses showed a significant main effect of condition [*F*_(5, 65)_ = 3.91, *p* = 0.007, η^2^_*p*_ = 0.231] and a marginally significant interaction between condition and location [*F*_(20,260)_ = 2.22, *p* = 0.052, η^2^_*p*_ = 0.146]. Further analysis revealed that ERPs elicited by new words were more negative than ERPs elicited by old words encoded in the positive low-arousing (*p*s < 0.05) and positive high-arousing contexts (*p*s < 0.05) at fronto-central, central, centro-parietal, and parietal locations, by old words encoded in the negative low-arousing (*p*s < 0.05) and neutral contexts (*p*s < 0.05) at central, centro-parietal, and parietal locations, and by old words encoded in the negative high-arousing context at parietal location (*p* < 0.05). In addition, our analyses indicated that ERPs elicited by old words encoded in the positive high-arousing context were more positive than ERPs elicited by old words encoded in the negative high-arousing context at frontal, central, and centro-parietal locations (*p*s < 0.05). ERPs elicited by old words encoded in the positive low-arousing context also were more positive than ERPs elicited by old words encoded in the negative high-arousing context at frontal, fronto-central, central, and centro-parietal locations (*p*s < 0.05).

***700–1000 ms***. Our analyses showed a marginally significant main effect of condition [*F*_(5, 65)_ = 2.66, *p* = 0.059, η^2^_*p*_ = 0.170] and a significant interaction between condition and location [*F*_(20,260)_ = 4.15, *p* = 0.001, η^2^_*p*_ = 0.242]. Further analysis revealed that ERPs elicited by new words were more negative than ERPs elicited by old words encoded in the positive high-arousing context at frontal, fronto-central, central, and centro-parietal locations (*p*s < 0.05). In addition, our analyses indicated that ERPs elicited by old words encoded in the positive high-arousing context were more positive than ERPs elicited by old words encoded in the neutral context at fronto-central, central, centro-parietal, and parietal locations (*p*s < 0.05), by old words encoded in the negative high-arousing (*p*s < 0.05) and negative low-arousing contexts (*p*s < 0.05) at all analyzed locations, and by old words encoded in the positive low-arousing context at frontal, central, and centro-parietal locations (*p*s < 0.05).

## Discussion

The present study explored the influence of emotional background pictures on memory for neutral words superimposed centrally onto them. The behavioral results indicate significant effects of emotional contexts. The negative high-arousing, negative low-arousing, and positive high-arousing contexts all impaired recognition performance for words as compared to neutral context. The finding that emotional contexts impaired memory for neutral items is consistent with some previous studies using the serial presentation design in which both emotional and neutral items were test items (e.g., Strange et al., 2003; Hurlemann et al., 2005; Knight and Mather, 2009). Our results suggest that emotional contexts can impair memory for neutral items even if they are task-irrelevant. In contrast to the current results, some studies did not find similar impairment (e.g., Smith et al., 2004; Jaeger et al., 2009). As said in the introduction, in the studies of Smith et al. (2004) and Jaeger et al. (2009), participants were asked to imagine a connection between the item and the background in the study phase. This instruction encouraged participants to represent the item and the context in a unitized manner. Thus, emotion-induced impairment of memory was not observed. Moreover, there were only two levels (positive and negative) of encoding contexts in the study of Erk et al. (2005), in which they did not introduce the neutral context as a baseline, making it difficult to find significant effect of encoding context on recognition performance. In the study by Kim et al. (2013), no impairment effect for central items was found probably because these items were part of picture stories.

The present study also showed that participants' responses were less accurate for words learned in the negative and positive high-arousing contexts as compared to words learned in the positive low-arousing context. However, no significant difference between words encoded in the neutral context and words encoded in the positive low-arousing context was found. These results suggest that effect of emotional contexts on memory for neutral items depends upon the type of emotional contexts. Compared with the neutral context, the negative contexts and the positive high-arousing context, rather than the positive low-arousing context, can impair memory for neutral items. In addition, participants' responses were more accurate for words learned in the positive low-arousing context than those learned in the positive high-arousing context. That is, the present study showed an arousal effect for positive contexts. Because low-arousing and high-arousing pictures were not completely matched on valence ratings, we cannot ensure that this arousal effect has not been confounded with the valence difference at all.

Besides recognition performance, the present study analyzed the ERP data. At encoding, compared with items presented in the neutral context, items in the positive and negative high-arousing contexts elicited more positive ERPs from 150 to 1000 ms post-stimulus. At same time window, significant arousal effects also were observed. That is, items in the positive high-arousing context elicited more positive ERPs than those in the positive low-arousing context, and items in the negative high-arousing context elicited more positive ERPs than those in the negative low-arousing context. The previous studies on ERP correlates of emotional stimulus processing have linked the earlier ERP components (<300 ms) to attention orientation for emotional stimuli and suggested that the later ERP components (>300 ms) reflect mental resource allocation and memory formation (see Olofsson et al., 2008). And many studies have showed that emotional arousal is relevant for attention capture and cognitive resource allocation during information processing (Lang et al., 1993; Schupp et al., 2003, 2007; Kissler et al., 2007). Following this line of thinking, we thought that in the present study the earlier effects of emotion and arousal might reflect an automatic process of attention capturing for high-arousing contexts, and the late effects might reflect a conscious and effortful process of overcoming the interference of high-arousing contexts. Of course, we acknowledge that these two processes are not definitive based on ERP results alone.

In addition, from 300 to 1000 ms post-stimulus, significant valence effect was observed in the high-arousing conditions. The items in the positive high-arousing context elicited more positive ERPs than those in the negative high-arousing context, which suggests that, after 300 ms post-stimulus, overcoming the interference of positive high-arousing context to items rehearsal might be more difficult as compared to the negative high-arousing context.

A recent study (Uribe et al., 2011) primarily examined the encoding of visual stimuli in either emotionally arousing or neutral contexts created by delivering auditory information during the presentation of the fixation cross between the visual stimuli. The authors found that emotional context modified the evoked potentials (larger negativity) from 80 to 140 ms after visual stimuli onset. Moreover, evoked theta and gamma power were higher in the emotional context. The authors suggested that visual encoding in emotional context is facilitated by mechanisms of expectancy, attention, and sensory processing that are recruited by the context itself. Obviously, our results differed from those of Uribe et al. (2011). However, this inconsistency is not surprising because in Uribe et al. (2011) study emotionally arousing stimuli and target stimuli were presented in different sensory modalities.

The current study also attempted to determine which ERP component during retrieval was influenced by emotional encoding context. The process of recognition can include two components: one is automatic recognition process driven by familiarity, and the other is the retrieval of details associated with a previously experienced event (i.e., recollection) (Mandler, 1980; Jacoby, 1991; Curran, 2000; Yonelinas, 2002; Curran et al., 2006; Rugg and Curran, 2007). Previous research has showed that the FN400 is an ERP component related to familiarity (Curran, 2000; Rugg and Curran, 2007) although whether FN400 reflects familiarity is still under debate (Paller et al., 2007; Hou et al., 2013), and the LPC is mainly associated with recollection (Paller, 2000; Rugg and Allan, 2000; Curran et al., 2006). Similar to these previous studies, the present study found two obvious old/new effects in ERPs: the FN400 effect during 300–400 ms and the LPC effect mainly at parietal site during 400–600 ms. New words elicited a larger FN400 and a smaller LPC than old words. Our behavioral results also showed significant old/new effects. Participants' responses were more accurate for correctly rejected new words than for correctly recognized old words, which was consistent with previous studies (Maratos and Rugg, 2001; Jaeger et al., 2009).

More importantly, our ERP results revealed the influence of emotional context on retrieval. During 300–400 ms, new words elicited a larger FN400 as compared to old words learned in the negative low-arousing, positive low-arousing, positive high-arousing, and neutral contexts. That is, except for the negative high-arousing condition, the FN400 old/new effects occurred under all other conditions. Moreover, old words learned in the negative high-arousing context elicited a larger FN400 than those learned in the positive high-arousing and low-arousing contexts. Previous studies have shown that FN400 mainly reflects an automatic recognition process that is driven by familiarity (Rugg and Curran, 2007). In this process, participants recognized a stimulus that had been experienced without consciously retrieving the relevant details or background information associated with that learned stimulus. Our ERP results suggest that negative high-arousing encoding context might impair the familiarity process. However, because some researchers have suggested that FN400 indicates conceptual priming rather than familiarity (Paller et al., 2007; Hou et al., 2013), we must treat this inference with caution.

A plausible explanation for the lack of the FN400 old/new effect in the negative high-arousing condition was that the negative high-arousing pictures can capture attention more effectively in the earlier stage of visual processing (Dolcos and Cabeza, 2002; Schupp et al., 2003; Delplanque et al., 2004; Carretie et al., 2006) than other pictures and thus impact the encoding of the words, especially when the word presentation was quite brief. Additionally, after participants' attention was captured by the negative high-arousing background, they might tend to avoid the negative high-arousing background because the present study used images of bloody scenes as negative high-arousing backgrounds. Because of the simultaneous presentation of the words and emotional backgrounds, avoidance from the negative high-arousing backgrounds might result in avoidance from words presented in these contexts and thus impaired the processing of those words in different aspects, such as perception, elaborative encoding, and rehearsal. As a result, during recognition participants would experience a lower level of familiarity with those words encoded in the negative high-arousing context.

For the 400–600 ms interval during recognition, we found significant LPC old/new effects in all conditions of emotional contexts. However, scalp distribution of LPC old/new effects in different conditions of emotional contexts was different. The LPC old/new effects in the positive low-arousing and high-arousing contexts occurred at fronto-central, central, centro-parietal, and parietal locations. The LPC old/new effects in the negative low-arousing and neutral contexts occurred at central, centro-parietal, and parietal locations. The LPC old/new effect in the negative high-arousing context occurred only at parietal location. And ERPs elicited by old words encoded in the positive high-arousing and low-arousing contexts were more positive than ERPs elicited by old words encoded in the negative high-arousing context at from frontal to centro-parietal locations. In other words, the LPC old/new effect in the negative high-arousing condition was smaller than that in the positive high-arousing and low-arousing conditions. Previous studies have linked the LPC old/new effect to the recollection process (Curran et al., 2006; Rugg and Curran, 2007; Woroch and Gonsalves, 2010). In this process, people attempt to recall an item along with details associated with the encoding context. Thus, the present results suggest that emotional encoding context might have impact on participants' recollection. People might recollect more information in the positive high-arousing and low-arousing conditions than in the negative high-arousing condition.

In the present study, a late old/new effect (i.e., old words elicited a more positive LSW than new words) were also found in the positive high-arousing condition during 700–1000 ms post-stimulus at from frontal to centro-parietal locations. Moreover, in this interval, words learned in the positive high-arousing context elicited a more positive LSW than those learned in other contexts. Maratos and Rugg (2001) found that the right frontal old/new effect was elicited solely by words studied in negative sentences during 1100–1944 ms. In the study of Smith et al. (2004), the most obvious ERP effect of emotional (positive and negative) contexts (vs. neutral context) appeared after about 800 ms post-stimulus. Inconsistent with these previous studies, the present study did not find a late old/new effect in the negative high-arousing condition. However, we agreed with Maratos and Rugg (2001) and Smith et al. (2004) on that this late emotion effect reflects an emotional modulation of post-retrieval processing such as evaluation and monitoring operations. Following this line of thinking, our results might reflect more retrieving for positive high-arousing contextual information and subsequent evaluation and monitoring processing.

As mentioned earlier, one limitation of the present study is that low-arousing and high-arousing pictures were not matched on valence ratings and positive and negative pictures were not matched on arousal ratings. Therefore, we cannot rule out the possibility that the effects of arousal and valence observed in the present study might be confounded to some extent. Another limitation of the present study is that the sample size was low. Although we had sufficient power to detect the influences of emotional context based *p*-values and effect size data provided in the result section, future studies with larger sample size are still need to help support the conclusions made in the present study.

In conclusion, the present study used the behavioral and ERP measurements to investigate the influences of different types of emotional encoding contexts on the memory process of neutral words. Behavioral results showed that words learned in the neutral context had higher recognition accuracy than those learned in the negative high-arousing, negative low-arousing, and positive high-arousing contexts. In the meantime, ERP results demonstrated the influences of emotional encoding contexts on the encoding and retrieval processes. At encoding, compared with items presented in the neutral context, items in the positive and negative high-arousing contexts elicited more positive ERPs from 150 to 1000 ms post-stimulus, which might reflect an automatic process of attention capturing of high-arousing context and a conscious and effortful process of overcoming the interference of high-arousing context. During retrieval, significant FN400 old/new effects occurred in conditions of negative low-arousing, positive low-arousing, positive high-arousing, and neutral contexts but not negative high-arousing context. Significant LPC old/new effects occurred in all conditions of encoding contexts. However, the LPC old/new effect in the negative high-arousing condition was smaller than that in the positive high-arousing and low-arousing conditions. These results suggest that emotional encoding context might influence both the familiarity and recollection processes. However, because of the lack of behavioral measures of these processes, our inferences should be considered tentative. In addition, during 700–1000 ms post-stimulus, a late old/new effect was found only in the positive high-arousing condition, which might reflect emotional modulation of post-retrieval processing.

## Author contributions

Qin Zhang co-designed the experiment, analyzed the data, created the figures, and co-wrote the text. Xuan Liu co-designed the experiment, co-collected the data, and co-wrote the text. Wei An co-wrote the text and advised on many aspects of the research. Yang Yang co-collected the data and advised on many aspects of the research. Yinan Wang co-wrote the text.

### Conflict of interest statement

The authors declare that the research was conducted in the absence of any commercial or financial relationships that could be construed as a potential conflict of interest.

## Supplementary material

The Supplementary Material for this article can be found online at: http://www.frontiersin.org/journal/10.3389/fnhum. 2015.00073/abstract
