# Supplementary material for: Recognition memory of neutral words can be impaired by task-irrelevant emotional encoding contexts: behavioral and electrophysiological evidence
Source: Front Hum Neurosci. 2015 Feb 13;9:73. doi: 10.3389/fnhum.2015.00073 (PMC4327741; doi:10.3389/fnhum.2015.00073)
Supplement: Supplementary file 1 [file Presentation1.PDF]

Supplementary Material

IAPS number of negative low-arousing pictures:
1270, 1275, 1280, 2100, 2110, 2205, 2276, 2312, 2490, 2590, 2682, 2700, 2710, 2722, 2750, 2900, 3300, 4621, 6010, 6020, 6241, 9000, 9001, 9007, 9008, 9041, 9045, 9046, 9090, 9101, 9102, 9110, 9180, 9220, 9280, 9290, 9330, 9331, 9340, 9373, 9390, 9404, 9415, 9421, 9440, 9530, 9561, 9584, 9830, 9912

IAPS number of negative high-arousing pictures:
1052, 1111, 1300, 2120, 2352.2, 3000, 3010, 3015, 3030, 3051, 3053, 3060, 3061, 3062, 3063, 3064, 3071, 3080, 3100, 3102, 3110, 3120, 3130, 3140, 3150, 3160, 3168, 3170, 3261, 3266, 3400, 3550, 6230, 6250, 6260, 6312, 6313, 6350, 6360, 6510, 6540, 6550, 6560, 6570, 7361, 8230, 9300, 9405, 9570, 9921

IAPS number of positive low-arousing pictures:
1340, 1460, 1463, 1601, 1603, 1604, 1610, 1620, 1721, 1750, 1920, 2040, 2057, 2070, 2080, 2165, 2260, 2311, 2331, 2340, 2341, 2360, 2370, 2391, 2530, 2550, 2650, 2660, 5000, 5001, 5010, 5200, 5201, 5220, 5300, 5594, 5760, 5780, 5820, 5830, 5831, 5891, 5982, 7325, 7410, 7470, 7480, 7580, 8461, 8497

IAPS number of positive high-arousing pictures:
4001, 4002, 4003, 4180, 4210, 4220, 4232, 4235, 4240, 4290, 4300, 4310, 4320, 4460, 4470, 4490, 4520, 4520, 4530, 4561, 4599, 4601, 4606, 4607, 4608, 4609, 4611, 4640, 4641, 4650, 4651, 4652, 4653, 4656, 4658, 4659, 4660, 4664, 4666, 4669, 4670, 4672, 4680, 4681, 4683, 4687, 4689, 4690, 4800, 4810

IAPS number of neutral pictures:
1670, 2190, 2200, 2215, 2372, 2383, 2410, 2495, 2749, 2840, 2850, 2870, 5390, 6150, 7000, 7500, 7004, 7006, 7009, 7010, 7025, 7030, 7034, 7035, 7040, 7050, 7080, 7090, 7100, 7110, 7140, 7150, 7170, 7175, 7185, 7211, 7217, 7233, 7351, 7490, 7491, 7496, 7503, 7595, 7620, 7705, 7710, 7950, 9210, 9401
